# First- vs second-line CDK 4/6 inhibitor use for patients with hormone receptor positive, human epidermal growth-factor receptor-2 negative, metastatic breast cancer in the real world setting

**DOI:** 10.1007/s10549-024-07415-6

**Published:** 2024-06-26

**Authors:** Gretchen Kimmick, Asal Pilehvari, Wen You, Gloribel Bonilla, Roger Anderson

**Affiliations:** 1 Duke University Medical Center/Duke Cancer Institute, DUMC Box 3204, Durham, NC 27710 USA; 2 Department of Public Health Sciences, University of Virginia, Charlottesville, VA USA; 3 University of Virginia Comprehensive Cancer Center, Charlottesville, VA USA

**Keywords:** Metastatic breast cancer, Hormone receptor positive, Endocrine therapy, Real-world data, Treatment duration, CDK4/6 inhibitor

## Abstract

**Purpose:**

To compare CDK4/6 inhibitor (CDK4/6i) with endocrine therapy (ET) in the first- versus second-line setting for treatment of hormone receptor positive (HR+), HER2 negative, metastatic breast cancer (MBC) using real-world evidence.

**Methods:**

Patients with HR+, HER2 negative MBC, diagnosed between 2/3/2015 and 11/2/2021 and having ≥ 3 months follow-up were identified from the nationwide electronic health record-derived Flatiron Health de-identified database. Treatment cohorts included: (1) first-line ET with a CDK 4/6i (1st-line CDK4/6i) versus (2) first-line ET alone followed by second-line ET with a CDK4/6i (2nd-line CDK4/6i). Differences in baseline characteristics were tested using chi-square tests and two-sample t-tests. Time to third-line therapy, time to start of chemotherapy, and overall survival were compared using Kaplan-Maier method.

**Results:**

The analysis included 2771 patients (2170 1st-line CDK4/6i and 601 2nd-line CDK4/6i). Patients receiving 1st-line CDK4/6i were younger (75% vs 68% < 75 years old, p = 0.0001), less likely uninsured or not having insurance status documented (10% vs. 13%, p = 0.04), of better performance status (50% vs 43% with ECOG 0, p = 0.03), and more likely to have de novo MBC (36% vs. 24%, p < 0.001). Time to third-line therapy (49 vs 22 months, p < 0.001) and time to chemotherapy (68 vs 41 months, p < 0.001) were longer in those receiving first-line CDK4/6i. Overall survival (54 vs 49 months, p = 0.33) was similar between groups.

**Conclusion:**

Use of CDK4/6i with first-, vs second-, line ET was associated with longer time to receipt of 3rd-line therapy and longer time to receipt of chemotherapy.

## INTRODUCTION

Female breast cancer is the most common cancer in the U.S. and other developed countries, with the hormone receptor positive (HR+), HER2 negative subtype (HR + BC) comprising approximately 70% of diagnosed cases. While more than 85% of women diagnosed in the U.S. survive 5 years or more with primary therapy for non-metastatic disease, women diagnosed with metastatic breast cancer (MBC) have a much poorer prognosis, with less than one-third surviving 5 years. Cyclin dependent kinase 4/6 inhibitors (CDK4/6i) are a recently approved class of drugs (February 2015) that target enzymes important in cell division and thus can interrupt the growth of cancer cells [1]. This is important for breast cancer because the cyclin D/cyclin-dependent kinases 4 and 6 (CDK4/6)–retinoblastoma protein (RB) pathway plays a key role in the proliferation of both normal breast epithelium and breast cancer cells. The genes repressed by CDK4/6 inhibition are strongly associated with clinical prognosis in HR + BC [2].

There are currently three CDK4/6i drugs FDA-approved for the treatment of HR + MBC: abemaciclib (Verzenio), palbociclib (Ibrance) and ribociclib (Kisqali). Large randomized, phase III trials have consistently shown improvements in progression-free survival (PFS) with the addition of CDK4/6i to endocrine therapy (ET) in the 1st-line setting with an aromatase inhibitor [3–11] and 2nd-line setting with fulvestrant [12–15]. Across trials, the hazard ratio for progression-free survival (PFS) is approximately 0.5 (0.46–0.59). With longer follow-up, we are also seeing benefits in terms of overall survival (OS) for treatment in either the first- or second-line setting [16–19]. Likewise, real-world comparative effectiveness research mirrors results of clinical trials. Studies using FlatIron Health Database show longer progression-free survival (PFS), longer time to starting chemotherapy, and longer time to third-line therapy with palbociclib + ET versus ET alone as the 1st-line therapy [20–23]. Results also held true for improved PFS with palbociclib in the 1st-line setting among African American patients and patients with visceral metastases in the FlatIron Health Database [24, 25]. A large, single institution study from MD Anderson Cancer Center, including over 5000 patients, showed improved PFS when palbociclib was added to ET in the 1st- or 2nd-line settings and, importantly, better OS rates in the 2nd-line setting compared to ET alone [26]. In a SEER-Medicare population-based study, that included all three CDK4/6i, overall survival rates were improved with use of CDK4/6i plus ET versus ET alone [27].

Although CDK4/6i drugs are FDA approved for 1st-line and later therapy settings, because of overall survival benefits, guidelines now recommend that they be included as part of 1st line therapy, with the caveat that there are postmenopausal women for whom endocrine monotherapy is appropriate [36]. The guidelines state that the decision to offer 1st line endocrine monotherapy should be based on low disease burden, long disease-free interval, patient age, patient choice and other factors, including treatment tolerance. No validated markers exist to allow us to choose patients who might avoid the high expense of adding a CDK4/6i to their 1st-line ET. Recent results from the SONIA trial [28, 29] which tested the benefit of 1st-line CDK4/6i versus 2nd-line CDK4/6i by randomizing women with HR+/HER2 advanced breast cancer to either (1) 1st-line aromatase inhibitor + CDK4/6i followed by 2nd-line fulvestrant alone or (2) 1st-line aromatase inhibitor alone followed by 2nd-line CDK4/6i + fulvestrant. The SONIA trial found no benefit in second PFS, OS, or health-related quality of life to including CDK4/6i in the 1st-line. However, no study has yet compared real-world outcomes in patients who receive 1st-line versus 2nd line CDK4/6i. This study fills this gap by analyzing sequence of CDK4/6i use in the real-world setting using the Flatiron Health Database and including any CDK4/6i with any ET, to to compare outcomes for patients receiving 1st versus 2nd line CDK4/6i with ET.

## METHODS

### Data

Patients were derived from the nationwide electronic health record (EHR)-derived Flatiron Health de-identified database. The Flatiron Health database is a longitudinal database, comprising de-identified patient-level structured and unstructured data, curated via technology-enabled abstraction [30, 31]. During the study period, the de-identified data originated from approximately 280 US cancer clinics (~ 800 sites of care). The majority of patients in the dataset originate from community oncology settings; relative community/academic proportions may vary depending on the study cohort. The data are de-identified and subject to obligations to prevent re-identification and protect patient confidentiality.

The study included 2771 patients diagnosed with HR+, HER2 negative, MBC; who had 3 months of follow-up after the date of metastatic diagnosis date (Index date) beginning from 03 February 2015 to 02 November 2021 (see Appendix), and who received ET with a CDK 4/6i in the 1st-line treatment setting (1st-line CDK4/6i) or ET alone in the 1st-line setting and then ET with a CDK4/6i in the 2nd-line setting (2nd-line CDK4/6i). Line of therapy was defined based on sequence of therapy after Index date, as previously described [32] and used in other similar work [33]. Patients were excluded if they had a first structured activity (vital records, a medication administration, or a laboratory test/result) more than 90 days after the index date; received prior treatment with CDK4/6i (palbociclib, abemaciclib, ribocliclib); received first line therapy more than 30 days before the metastatic diagnosis date. We then further limited the study population to those who received ET with a CDK4/6i either in first-line setting, or in the second-line setting, after receiving ET alone in the first-line setting. Eligibility for inclusion in the study sample was not limited by receipt or type of therapy in the 2nd-line therapy in those patients who received 1st-line ET + CDK4/6i.

### Outcomes

Comparative analyses were conducted to investigate the impact of the timing of receiving CDK 4/6i (first-line versus second-line) on real-world time to third line therapy (1st primary outcome), time to chemo therapy (2nd primary outcome), and real-world overall survival (secondary outcome).

The time to third line therapy starts from the start of first line therapy to the start of third line therapy or death. The exact start date of each therapy line is available in the database. Patients who did not die or have the third line therapy were censored at their last structured visit date within the study time frame. Time to chemotherapy is defined similar to the third line therapy (List of chemotherapy drugs are provided in Appendix).

**Table 1:** Patient’s characteristics by subgroups

	Overall population	1st-line CDK4/6i	2nd-line CDK4/6i	p (comparing difference between groups of 1st and 2nd line CDK 4/6i)
Age				0.0001
18–49	141 (5.1)	92 (4.2)	49 (8.2)	
50–64	936 (33.8)	762 (35.1)	174 (29.0)	
65–74	960 (34.6)	774 (35.7)	186 (30.9)	
75+	734 (26.5)	542 (25.0)	192 (31.9)	
Total	2771 (100)	2170 (100)	601 (100)	
Race/ethnicity				0.15
Asian	52 (1.9)	42 (1.9)	10 (1.7)	
Black	238 (8.6)	183 (8.4)	55 (9.2)	
Hispanic or Latino	199 (7.2)	154 (7.1)	45 (7.5)	
Not documented	241 (8.7)	205 (9.4)	36 (6.0)	
Other Race	216 (7.8)	163 (7.5)	53 (8.8)	
White	1825 (65.9)	1423 (65.6)	402 (66.9)	
Total	2771 (100)	2170 (100)	601 (100)	
Heath insurance type at time between diagnosis of metastases and first line treatment				0.16
Commercial/Medicaid	63 (2.3)	49 (2.3)	14 (2.3)	
Commercial	467 (16.9)	384 (17.7)	83 (13.8)	
Commercial/Other	266 (9.6)	212 (9.8)	54 (9.0)	
Medicaid only	33 (1.2)	26 (1.2)	7 (1.2)	
Medicare	372 (13.4)	280 (12.9)	92 (15.3)	
Medicare/Medicaid	89 (3.2)	68 (3.1)	21 (3.5)	
Medicare/other	1196 (43.2)	941 (43.4)	255 (42.4)	
None	285 (10.3)	210 (9.7)	75 (12.5)	
Total	2771 (100)	2170 (100)	601 (100)	
Health Insurance status				0.04
Uninsured/not documented	285 (10.3)	210 (9.7)	75 (12.5)	
Insured	2486 (89.7)	1960 (90.3)	526 (87.5)	
Total	2771 (100)	2170 (100)	601 (100)	
Year starting CDK4/6i				< 0.001
2015	148 (5.3)	126 (5.8)	22 (3.7)	
2016	295 (10.6)	233 (10.7)	62 (10.3)	
2017	412 (14.9)	292 (13.5)	120 (20.0)	
2018	509 (18.4)	398 (18.3)	111 (18.5)	
2019	518 (18.7)	402 (18.5)	116 (19.3)	
2020	488 (17.6)	401 (18.5)	87 (14.7)	
2021	398 (14.4)	318 (14.7)	80 (13.3)	
2022	3 (0.1)	0 (0)	3 (0.5)	
Total	2771 (100)	2170 (100)	601 (100)	
ET backbone				< 0.001
Anastrozole	–	211 (9.7)	63 (10.5)	
Anastrozole, Fulvestrant	–	6 (0.3)	26 (4.3)	
Exemestane	–	46 (2.1)	20 (3.3)	
Exemestane, Fulvestrant	–	6 (0.3)	4 (0.7)	
Fulvestrant	–	604 (27.8)	254 (42.3)	
Letrozole	–	1277 (58.9)	213 (35.4)	
Letrozole, Fulvestrant	–	12 (0.6)	14 (2.3)	
Tamoxifen	–	7 (0.3)	1 (0.2)	
Tamoxifen, Fulvestrant	–	1 (0.1)	6 (1.0)	
Total	–	2170 (100)	601 (100)	
Backbone CDK4/6i				0.33
Abemaciclib	–	178 (8.2)	46 (7.7)	
Abemaciclib, Ribociclib	–	2 (0.1)	1 (0.2)	
Palbociclib	–	1793 (82.6)	503 (83.7)	
Palbociclib, Abemaciclib	–	24 (1.1)	1 (0.2)	
Palbociclib, Ribociclib	–	7 (0.3)	1 (0.2)	
Ribociclib	–	166 (7.7)	49 (8.2)	
Total	–	2170 (100)	601 (100)	
Duration of time on CDK4/6i mean (SD), months	18.9 (16.1)	19.8 (16.4)	15.2 (14.1)	0.001
ECOG PS				0.03
0	1179 (48.7)	959 (50.2)	220 (43.1)	
1	865 (35.7)	673 (35.2)	192 (37.6)	
2	287 (11.9)	213 (11.2)	74 (14.5)	
3, 4	89 (3.7)	65 (3.4)	24 (4.7)	
Not Documented				
Total	2420 (100)	1910 (100)	510 (100)	
Number of recorded comorbidities				0.17
0	1682 (60.7)	1320 (60.8)	362 (60.2)	
1	808 (29.2)	638 (29.4)	170 (28.3)	
2	256 (9.2)	197 (9.1)	59 (9.8)	
3	25 (0.9)	15 (0.7)	10 (1.7)	
Total	2771 (100)	2170 (100)	601 (100)	
Metastases presentation				< 0.001
De novo	925 (33.4)	779 (35.9)	146 (24.3)	
Relapsed	1846 (66.6)	1391 (64.1)	455 (75.6)	
Total	2771 (100)	2170 (100)	601 (100)	
Metastases location				0.2
Bone only	816 (29.4)	644 (29.7)	172 (28.6)	
Visceral	650 (23.5)	522 (24.1)	128 (21.3)	
Non-visceral	1305 (47.1)	1004 (46.3)	301 (50.1)	
Total	2771 (100)	2170 (100)	601 (100)	
Number of metastatic sites				0.30
1	1066 (38.5)	845 (38.9)	221 (36.8)	
2	755 (27.2)	598 (27.6)	157 (26.1)	
3	490 (17.7)	370 (17.1)	120 (20.0)	
4	275 (9.9)	208 (9.6)	67 (11.1)	
≥ 5	185 (6.7)	149 (6.9)	36 (6.0)	
Total	2771 (100)	2170 (100)	601 (100)	
Time from initial diagnosis to metastases, n (%), years				0.0001
De novo	925 (33.4)	779 (35.9)	146 (24.3)	
≤ 1	78 (2.8)	56 (2.6)	22 (3.7)	
> 1–5	533 (19.2)	351 (16.2)	182 (30.3)	
> 5	1229 (44.4)	979 (45.1)	250 (41.6)	
Not documented	6 (0.2)	5 (0.2)	1 (0.2)	
Total	2771 (100)	2170 (100)	601 (100)	

We defined overall survival (OS) as the number of months from the start of first-line therapy to death as provided in the Flatiron dataset. Patients who did not die were censored at the last date of structured activity.

### Other variables

Demographic variables, including age, race/ethnicity, and health insurance type (Commercial, Medicare, Medicaid, and etc.) before the start of first line therapy, and clinical characteristics, including ECOG performance status (PS), number of comorbidities, site of metastasis, stage of cancer at the initial diagnosis (I, II, III, IV), de novo versus relapse MBC, and the year of treatment initiation, were included in analyses.

### Statistical analysis

Chi-squared tests were performed to test the differences in baseline demographics and clinical characteristics between those who received ET with CDK4/6i as 1st-line therapy (1st-line CDK4/6i) versus those who received ET as 1st-line therapy followed by ET with a CDK4/6i as 2nd-line therapy (2nd-line CDK4/6i). When there was a significant difference in groups by chi-squared test, then two-sample statistical t-tests were used to compare within categories.

We conducted the Kaplan–Meier method and 95% confidence intervals to determine median values for the primary and secondary outcomes. To estimate hazard ratios and 95% confidence intervals for outcomes, Cox proportional regression analyses were performed. To tackle any observable differences in baseline characteristics including demographics and clinical traits between the two groups, we applied the Inverse Probability Weighting (IPW) method. Furthermore, Propensity Score Matching (PSM) was used as sensitivity analyses to check the robustness of the IPW method.

To overcome the issue of missing data, we incorporated a new category for "missing" values as an additional level in categorical variables, such as race and ECOG value, when the reason for the data being missing was not clear.

This study had approval from the Institutional Review Board at the University of Virginia.

## RESULTS

The study population included 2771 (70.7%) patients receiving 1st-line CDK4/6i (n = 2170) or 2nd-line CDK4/6i (n = 601). Demographic and clinical characteristics, shown in Table 1, differed between the two groups. Compared to patients receiving 2nd-line CDK4/6i, patients who received 1st-line CDK4/6i were younger (75% of the 1st-line CDK4/6i subgroup vs 68% in the 2nd-line CDK4/6i were less than 75 years old, p = 0.0001), were less likely to be uninsured or not have insurance status documented (10% vs. 13%, p = 0.04), had better performance status (50% vs. 43% patients with ECOG value 0, p-value = 0.03), and more likely to have presented with de novo MBC (36% vs. 24%, p-value < 0.001).

Most patients (59%) in 1st-line CDK4/6i were given letrozole as their ET backbone, while the majority of patients (42%) who received 2nd-line CDK4/6i were given fulvestrant as their ET backbone (p-value < 0.001). Patients who received 1st- line CDK4/6i were on CDK4/6i for significantly longer period compared to those who received 2nd- line CDK4/6i (20 vs. 15 months, p-value = 0.001). In the 1st-line CDK4/6i group, 983 received 2nd-line therapy: 37% continued ET + CDK4/6i but with a change in ET and/or CDK4/6i; 21% continued ET with another targeted agent (such as everolimus or a PIK3CA inhibitor); 18% continued ET alone; 23% received a chemotherapeutic agent; 1% received single-agent abemaciclib.

### Time to third-line therapy

**Fig. 1:**
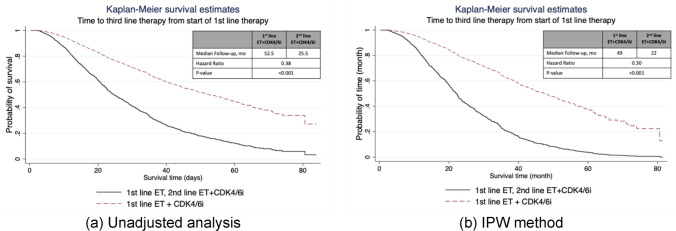
Kaplan–Meier curves of real-world time to third line therapy in (**a**) unadjusted analysis (**b**) IPW adjusted analysis

In the unadjusted analysis, median time to receipt of 3rd-line therapy was 52.5 months (95% CI, 48.3–56.9) for 1st-line CDK4/6i and 25.5 months (95% CI, 22.4–29.8) for 2nd-line CDK4/6i (p-value < 0.001, Fig. 1a). After IPW adjustment, time to 3rd-line was 49 months (95% CI,42.5–54.7) among patients treated with 1st-line CDK4/6i compared with 22 months (95% CI, 21.9–28.9) among patients treated 2nd-line CDK4/6i (hazard ratio, 0.30; p-value = 0.001; Fig. 1b). Sensitivity analysis using PSM method reports similar results.

### Time to chemotherapy

**Fig. 2:**
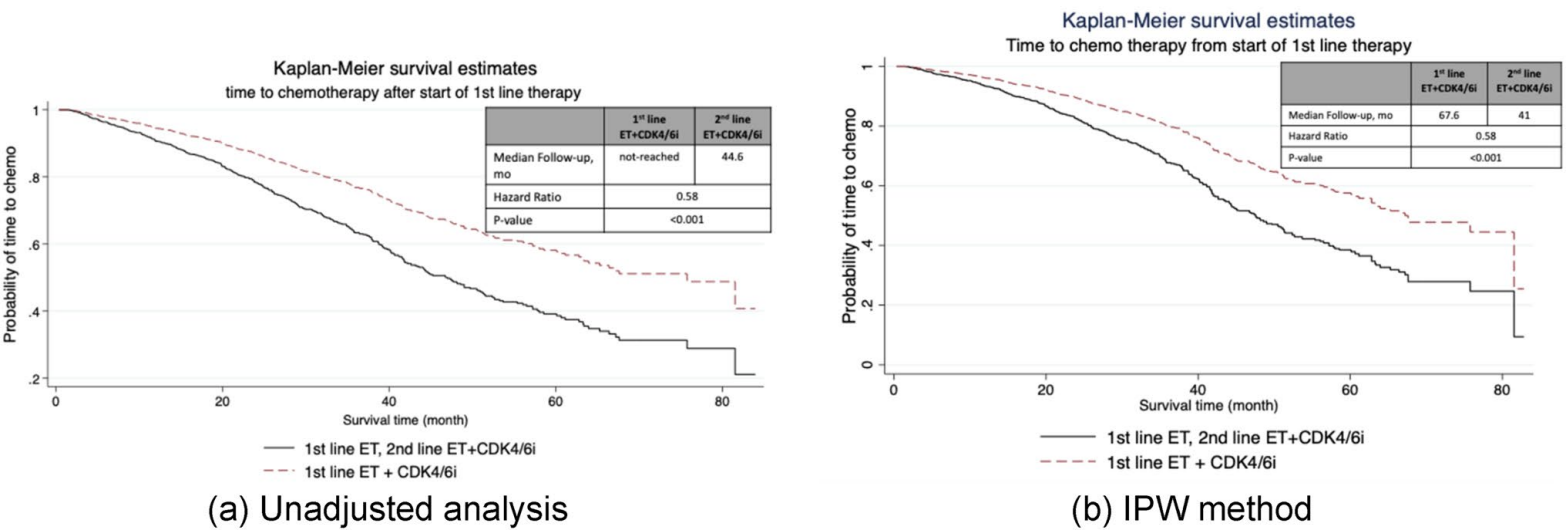
Kaplan–Meier curves of real-world time to chemotherapy in (**a**) unadjusted analysis (**b**) IPW adjusted analysis

Initiation of chemotherapy (capecitabine or IV chemotherapy) for treatment of metastatic disease was significantly different in patients who received 1st-line CDK4/6i versus those who received 2nd-line CDK4/6i (p-value < 0.001, Fig. 2). In the unadjusted analysis, among patients who received 2nd-line CDK4/6i, median time to chemotherapy was 44.6 months (95% CI, 39.1–50.6) and was not reached in patients who received 1st-line CDK4/6i (P < 0.0001; Fig. 2a). In IPW analysis, median time to start of chemotherapy for patients who received 1st-line CDK4/6i was 67.6 months, and for patients who received 2nd-line CDK4/6i was 41 months (p-value < 0.001, Fig. 2b).

### Overall survival

**Fig. 3:**
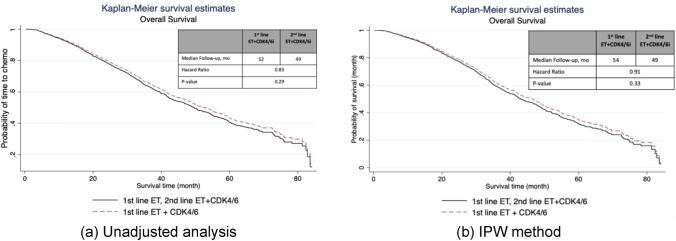
Kaplan–Meier curves of real-world overall survival in (**a**) unadjusted analysis (**b**) IPW adjusted analysis

Shown in Fig. 3, unadjusted analysis showed that median OS was 52 months (95% CI, 49.2–56.9) for patients receiving 1st-line CDK4/6i versus 49 months (95% CI, 44.1–54.7) for patient receiving 2nd-line CDK4/6i and was not significantly different between the groups (p = 0.29). Adjusted analysis revealed similar findings. In adjusted analysis, the median OS was 54 months (95% CI, 46.5–60.5) for patients who had 1st-line CDK4/6i versus 49 months (95% CI, 42.9–54.7) in those who received 2nd-line CDK4/6i, which are not statistically different (p-value = 33).

## DISCUSSION

In this real-world population of patients with HR+, HER2 negative, MBC, those with older age, lower PS, no insurance, and relapse after diagnosis and treatment for non-MBC were less likely to receive 1st-line CDK4/6i with ET. Compared to 1st-line CDK4/6i use, and after adjusting for patient characteristics, 2nd-line CDK4/6i use is associated with shorter time to 3rd-line therapy and shorter time to receipt of chemotherapy, though OS rates were similar. Prior evidence from real-world studies and randomized clinical trials demonstrates the importance of CDK4/6i use in patients with ER+, HER2 negative MBC, but there is limited data about the sequence of use. Randomized trials confirm longer PFS and OS when CDK4/6i is used with ET compared to ET alone, in the 1st- or 2nd-line setting, but do not control for what therapies are used after progression. Here, we chose patients receiving CDK4/6i with either 1st- or 2nd-line therapy, in order to determine if there was an advantage to having received a CDK4/6i with 1st-line ET or if it was acceptable to wait to prescribe it with 2nd-line ET. To our knowledge, we present the first real-world data to suggest that use of CDK4/6i in the 1st-line of ET may offer the benefit of longer time to chemotherapy than if CDK4/6i use is delayed to the 2nd-line of treatment.

Clinical trials have proven that adding CDK4/6i to ET compared to the use of ET alone in the treatment of HR+, HER2 negative MBC leads to improved rates of PFS and OS. In the 1st-line setting, phase III trials comparing an aromatase inhibitor with or without a CDK4/6i showed substantial improvements in PFS, with a consistent hazard ratio (HR) of approximately 0.5 [3, 5, 6, 8, 11], and OS with a HR of approximately 0.75 [16, 34]. In the second line setting, after 1st line single-agent AI, phase III trials showed improved PFS, with HR approximately 0.5 [10, 12, 14, 15], and OS, with HR approximately 0.8 [13, 19, 35]. Based on improved survival rates with the combination, consensus recommendations are that CDK4/6i be included in 1st-line ET for HR+, HER2 negative MBC [36]. Despite this advice, the added expense, monitoring, and the toxicity of CDK4/6i compared to ET alone has made some hesitate to use CDK4/6i in the 1st-line setting, delaying use of the CDK4/6i to 2nd-line, with the argument that we have no data to show that the survival advantage for use of CDK4/6i with ET versus ET alone is relevant to its use vs not or to the timing of its use. Since clinical trials, and previous real-world studies showing OS benefits for use of CDK4/6i + ET versus ET alone, did not control for what treatment was prescribed after progression, the OS benefit of CDK4/6i in 1st-line setting may have been amplified, compared to our real world study where both groups had received a CDK4/6i; in fact, many of the patients in the clinical trials may never have receive a CDK4/6i at all. This thought process, and the concern about toxicity and expense of CDK4/6i, provided the motivation to conduct the Selecting the Optimal positioN of CDK4/6 Inhibitors in hormone receptor-positive Advanced breast cancer (SONIA) trial and for our real-world study.

The SONIA trial was designed to define the optimal strategy for using CDK4/6i in clinical practice [28, 29]. In the SONIA trial, women with previously untreated HR+, HER2 negative metastatic breast cancer were randomized to receive a non-steroidal AI + CDK4/6i as 1st-line therapy and then switch to Fulvestrant on progression or to receive a non-steroidal AI as 1st-line therapy and switch to Fulvestrant + CDK4/6i on progression. Patients were monitored for progression every 12 weeks with a primary endpoint of time from randomization to second objective progression (PFS2), or time to 3rd-line therapy. Secondary endpoints included quality of life, OS, and cost-effectiveness. As compared to our report, where time to 3rd-line therapy was longer with 1st-line CDK4/6i, the time to 3rd-line therapy in the SONIA trial was not significantly different between study arms (31 months for 1st-line CDK4/6i versus 26.8 months for 2nd-line CDK4/6i; HR 0.87, 95% CI 0.74–1.03, p = 0.10). In the SONIA trial, OS was similar (HR 0.98, 95% CI 0.80–1.20, p = 0.83), as were quality of life measures, but toxicity and cost were greater with 1st-line CDK4/6i use. In subset analysis, patients who had not received prior adjuvant therapy (HR 0.78; 95% CI 0.64–0.97) and those with bone only disease (HR 0.64; 95% CI 0.42–0.98) benefited from receipt of 1st-line CDK4/6i. There are two important differences between our novel study of real-world practice compared to the SONIA trial. First, 1st-line ET was not limited to a nonsteroidal AI, which better reflects real-world practice where patients presenting with MBC who are taking an adjuvant AI would not be prescribed an AI at the time of progression. Second, in the group receiving 1st-line ET + CDK4/6i, we did not limit type of 2nd-line therapy to endocrine therapy alone. These difference in treatment patterns may explain the longer time to 3rd-line therapy and cytotoxic chemotherapy when CDK4/6i is added to ET in the 1st-line setting. Given the relative toxicity of chemotherapy, delaying its use is important for patients. The development of tumor resistance according to treatment sequence, however, must be considered and has yet to be fully studied.

In patients with ER+, HER2-negative MBC, ET is the cornerstone of the treatment [36]. Response rates to ET alone are high, but most patients experience cancer progression, likely due to the development of resistance to ET [37, 38]. Overactivity of the CDK4/6 pathway is common in patients with ER+, HER2-negative breast cancer and is one mechanism of ET resistance. CDK4/6i, therefore, helps overcome resistance to ET and prolongs time to disease progression and death. Data from clinical trials shows that the use of CDK4/6i prolongs PFS and OS. The SONIA trial showed that progression after two lines of therapy, survival, and quality of life are similar if women with untreated, HR+, HER2 negative MBC are treated with either non-steroidal AI + CDK4/6i followed by fulvestrant on progression or non-steroidal AI followed by fulvestrant + CDK4/6i on progression. The median time to 3rd-line therapy, however, was not reported, but the median duration on CDK4/6i was much longer for 1st-line CDK4/6i than for 2nd-line CDK4/6i (24.6 months vs 8.1 months) implying that development of resistance to CDK4/6i, and perhaps to ET, was delayed if CDK4/6i was used in the 1st-line setting. We look forward to more information when the SONIA trial results are peer-reviewed and published. This study, though retrospective, suggests that it is important to incorporate CDK4/6i with 1st-line therapy to prevent the development of ET resistance earlier in the treatment course and prolong time to chemotherapy. Our results diverge from SONIA in finding better median time to 3rd line therapy with 1st line CDK4/6i. This may be due to the fact that we did not restrict our analysis to class of ET treatment. Possible selection bias for 2nd line treatment is also important to consider. 

We found disparities in receipt of 1st-line CDK4/6i. Older age, lower PS, no insurance, and relapse after diagnosis and treatment for non-MBC were factors associated with receiving 2nd- instead of 1st- line therapy with CDK4/6i. Older age, poor PS, and lack of insurance are factors that have been associated with a lack of guideline-concordant therapy [39–41]. This is despite evidence showing that older patients tolerate treatment with CDK4/6i [42, 43]. We speculate that relapse after diagnosis and treatment for non-MBC is a factor because patients had already received adjuvant endocrine therapy, and were deemed responsive; thus, an aromatase inhibitor alone was offered as 1st-line therapy for MBC. In order to encourage optimal adherence to treatment guidelines calling for delivery of CDK4/6i in the 1st-line setting, particular attention should be paid to patients with older age, lower PS, no insurance, and relapse after diagnosis and treatment for non-MBC. This may be especially important, given the findings of the RIGHT Choice trial (Study to Compare the Combination of Ribociclib Plus Goserelin Acetate With Hormonal Therapy Versus Combination Chemotherapy in Premenopausal or Perimenopausal Patients With Advanced or Metastatic Breast Cancer) comparing 1st-line ET + CDK4/6i versus chemotherapy for pre- or peri-menopausal patients with HR+, HER2 negative “aggressive” MBC [44]. Patients receiving ET + CDK4/6i had similar time to treatment response, longer PFS, and fewer side effects than those who received chemotherapy, emphasizing the importance of offering ET + CDK4/6i to most patients with HR+, HER2 negative metastatic breast cancer.

The intrinsic weaknesses of using retrospective analysis of real-world data to draw conclusions about clinical care must be recognized. Use of retrospective data does not allow for control of numerous variables that might be important to the outcome, including volume of metastatic disease, comorbidity, and patient social support, to list a few. Fortunately, many demographic and clinical variables were available in the Flatiron database, which allowed us to control for possible confounders in our analysis. There may also be a bias created by the fact that 12% of the patients who received 1st-line therapy appeared to respond to ET for more than 5 years, which may indicate that the cohort of patients who received 2nd-line ET + CDK4/6i had more aggressive cancer and, therefore, worse outcomes. We were not able to control for this variable in our analysis.

This study also has several strengths. First, the Flatiron Database is large and has a wide geographic distribution, and sets a baseline for real-world patterns in 1st and 2nd line use of CDK4/6i, prior to SONIA data. Second, data within the Flatiron Database is prospectively collected, and provides detailed information about patient demographics and tumor characteristics, allowing adjustment of the analysis for baseline characteristics. Third, this database, unlike other administrative databases, includes information about disease status/progression, which is not available in most other databases, where only OS using National Death Index data might be available. We were, therefore, able to include rwPFS and OS. This methodology using the Flatiron Database has been validated by Bartlett et al., to compare RWE to data from randomized clinical trials [45]. Lastly, the OS endpoint from the Flatiron database has also been validated [46] and includes external data sources (NDI, US SS Death Index, obituaries, and commercial death data) in addition to health records.

In summary, we used a real-world database to compare characteristics and outcomes in patients receiving 1st-line ET + CDK4/6i for HR+, HER2 negative MBC versus those receiving 1st-line ET alone followed by 2nd-lineET + CDK4/6i and found that those receiving 1st-line ET + CDK4/6i had longer time to 3rd-line therapy and longer time to chemotherapy. However, OS was similar. The results of the SONIA trial, as presented at ASCO 2023, suggest that whether CDK4/6i is used with AI as 1st-line therapy or with fulvestrant as 2nd-line therapy, the PFS after those two lines of therapy is similar. In the absence of further randomized clinical trial data comparing these two treatment approaches, and until the SONIA [29] trial results are peer reviewed and published, our findings support the consensus guideline recommendations that CDK4/6i therapy should be offered with 1st-line ET [36].

## Data Availability

The datasets generated during and/or analyzed during the current are available for sharing upon request and approval from Flatiron.
